# Identifiability of structural networks of nonlinear electronic oscillators

**DOI:** 10.1038/s41598-020-71373-4

**Published:** 2020-09-04

**Authors:** V. P. Vera-Ávila, R. Sevilla-Escoboza, J. Goñi, R. R. Rivera-Durón, J. M. Buldú

**Affiliations:** 1 Centro Universitario de los Lagos, Universidad de Guadalajara, Enrique Díaz de Leon, Paseos de la Montaña, 47460 Lagos de Moreno, Jalisco Mexico; 2 Purdue Institute for Integrative Neuroscience, Purdue University, West-Lafayette, IN USA; 3 School of Industrial Engineering, Purdue University, West-Lafayette, IN USA; 4 Weldon School of Biomedical Engineering, Purdue University, West-Lafayette, IN USA; 5 Unmanned Systems Research Institute, Northwestern Polytechnical University, Xi’an, 710072 China; 6 Complex Systems Group and GISC, Universidad Rey Juan Carlos, Madrid, Spain; 7 Laboratory of Biological Networks, Center for Biomedical Technology, UPM, Pozuelo de Alarcón, 28223 Madrid, Spain

**Keywords:** Complex networks, Nonlinear phenomena

## Abstract

The interplay between structure and function is critical in the understanding of complex systems, their dynamics and their behavior. We investigated the interplay between structural and functional networks by means of the differential identifiability framework, which here quantifies the ability of identifying a particular network structure based on (1) the observation of its functional network and (2) the comparison with a prior observation under different initial conditions. We carried out an experiment consisting of the construction of $M=20$ different structural networks composed of $N=28$ nonlinear electronic circuits and studied the regions where network structures are identifiable. Specifically, we analyzed how differential identifiability is related to the coupling strength between dynamical units (modifying the level of synchronization) and what are the consequences of increasing the amount of noise existing in the functional networks. We observed that differential identifiability reaches its highest value for low to intermediate coupling strengths. Furthermore, it is possible to increase the identifiability parameter by including a principal component analysis in the comparison of functional networks, being especially beneficial for scenarios where noise reaches intermediate levels. Finally, we showed that the regime of the parameter space where differential identifiability is the highest is highly overlapped with the region where structural and functional networks correlate the most.

## Introduction

In the last decade, Neuroscience is one of the fields that has benefited the most from Network Science^1^, borrowing techniques and methodologies to describe structural and dynamical properties of the brain^2,3^. However, the other way around, consisting of generalizing neuroscience methods and transcend other applications where systems are organized in networks, is not so common. More recently, the concept of brain connectivity fingerprints has become a key research area^4–12^;

Here, we propose the generalization of the concept of brain connectivity fingerprints^4^ and, in particular, of *differential identifiability*^12^ to networks of dynamical oscillators, with the aim of better understanding the boundaries that hinder the identification of an underlying network structure from the observation of the dynamics of its nodes. In computational neuroscience and brain connectomics, the idea of identifiability consists on, given the functional connectome of one subject, to identify which functional connectome from a set belongs to that same subject. With this aim, a test set of functional connectomes $\{A_i^{test}\}$ (with $i=1,2,\ldots ,M$) is obtained from a group of *M* individuals using a brain imaging technique, such as functional magnetic resonance (fMRI), magnetoencephalography (MEG) or electroencephalography (EEG). Next, a second set (retest) of functional connectomes $\{A_i^{retest}\}$ is obtained. Comparison between functional connectomes is usually estimated by the Pearson’s correlation coefficient of the entire connectivity profiles. Based on this, two different fingerprinting measures have been proposed. Differential identifiability^12^ quantifies, on average, how much more similar are the functional connectomes of the same subjects (when comparing test and retest) with respect to how similar are functional connectomes of different subjects in the dataset. The higher the differential identifiability, the more identifiable are the subjects in the test–retest dataset, and hence the more fingerprints are present in the data. This fingerprinting score is a more continuous approximation to the concept of brain fingerprints than identification rate^4^. Identification rate quantifies, on average, how often (i.e. success rate) a functional connectome of a subject in the test set is the most similar to the functional connectome of the same subject in the retest set and viceversa.

The identifiability framework is based on group-level principal component analysis of functional connectomes that maximizes the abovementioned differential identifiability score. Such framework has been shown to uncover functional connectome fingerprints within and across sites, for a variety of fMRI tasks, over a wide range of scanning length, and with and without global signal regression^12,13^. Additionally, it has been shown that it provides more robust and reliable associations between connectivity and cognition^14^ as well as with disease progression in neurodegeneration^15^. Finally, it has been recently assessed the positive effect of such framework on uncovering fingerprints on network measurements derived from functional connectomes^16^.

When applying those concepts to the assessment of structural networks where nodes are nonlinear oscillators, it is worth noting that identifiability relies on two key requirements: (1) the dynamics of each structural network and, in turn, its corresponding functional networks, need to be similar when the experiment is repeated and, (2) each structural network must show different dynamics from the other networks.

Regarding requirement (1), identifiability is strongly connected to the concept of *consistency*. In the general context of nonlinear dynamics, a consistent system^17–19^ is one that can reproduce the same dynamics when the same external input is applied, no matter what the initial conditions of the dynamical system are. Consistency has been reported in physical systems, such as lasers^20,21^ or optoelectronic systems^22^, but also in biological systems, such as neurons^23^ or brain dynamics^19^. However, identifiability differs from consistency in the fact that it does not require the existence of external input. Furthermore in the context of functional networks, identifiability relies on maintaining the same amount of coordination/communication between all dynamical units conforming the whole system, leading to similar functional networks, no matter what the particular dynamics of the units are. One of the many issues in determining whether a set of dynamical systems are identifiable is how to quantify the level of similarity between the set of original functional networks and the “re-tested” one. As shown by Amico et al.^12^, the use of principal component analysis (PCA) is strongly recommended when dealing with experimental signals, where a certain amount of noise is contained in the time series and in the resulting connectivity estimations to be analyzed. A part of enhancing the level of identifiability, PCA allows determining what principal components of the functional networks should be filtered, not only for the analysis of the system’s identifiability, but also for the evaluation of other properties of the functional networks.

Importantly, the identifiability of subjects on brain connectivity datasets deals with two significant drawbacks, i.e., non-stationary levels of synchronization between brain regions and the presence of highly varying (within session and across subjects) non-stationary noise. Both limitations can not be tuned or controlled in brain imaging experiments. Hence it is not possible to evaluate and assess how increasing or decreasing the synchronization or functional coupling between nodes affects identifiability, or to what extent is noise inducing or compromising identifiability. To investigate these questions, in this paper, we analyzed how identifiability is related to the synchronization level of a functional network and the distortion introduced by noise. We carried out experiments with networks of nonlinear electronic oscillators, modifying the coupling strength between oscillators and assessing the synchronizability as a function of the level of synchronization. Besides, we evaluated the consequences of introducing different levels of noise in the construction of the functional networks and quantified how noise affected the estimated identifiability. Finally, we showed the benefits of introducing PCA in the evaluation of the correlation between functional networks and, ultimately, in the identifiability of the whole system.

## Results

### Identifiability of networks of dynamical systems

**Figure 1:**
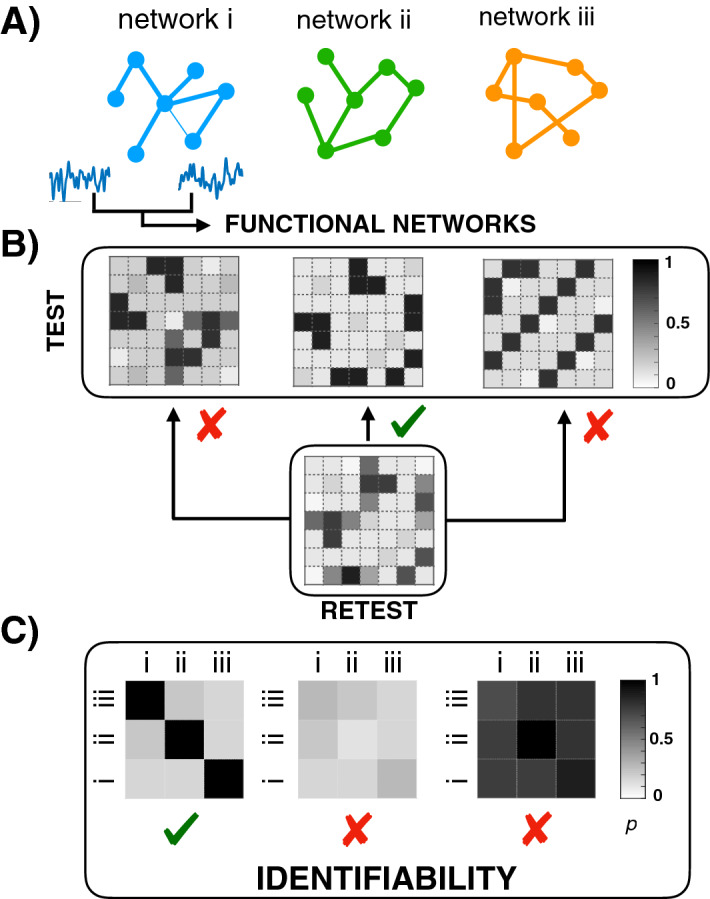
Schematic representation of the identifiability phenomenon. (**A**) The dynamics of the nodes of three different structural networks (i, ii, and iii) are recorded (“test”). (**B**) The coordination between all pairs of nodes is quantified, and the corresponding functional networks are obtained. In the figure, we plot the matrices containing the weights of the links of the functional networks. Next, a second measurement is carried out (“re-test”), and the corresponding functional network is compared with the previous ones. (**C**) All test (rows) and re-test (columns) functional networks of each structure (i,ii and iii) are compared by pairs. The elements of the matrix contain the correlations (*p*) between each pair of functional networks. Identifiable systems are those whose diagonal has much higher values than the off-diagonal ones (first matrix of the plot). On the contrary, when all values are low (second matrix) or high (third matrix), the identification is not possible.

The advantage of using electronic circuits to study identifiability is that the whole system can be configured in a desired way and all variables can be accessed, two facts that are imposible in the context of neuroscience. For this reason, it is important to detail all steps made during the construction of the system to be studied and how identifiability was measured. Figure 1 describes the procedure we followed to determine whether a network is identifiable given a group of different structures. First, the dynamics of a set of *M* structural networks were recorded ($M=3$ in the example of Fig. 1A). We called “test” to the set of first measurements of the time series of all dynamical units of each structural network. Second, the (weighted) adjacency matrices of the corresponding functional networks were obtained by quantifying the synchronization between oscillators. Third, the dynamics were recorded again and the second step was repeated in order to have a set of “re-test” functional networks. Finally, the test and re-test functional networks are compared in order to decide whether a structural network can be recognized from the comparison of its functional networks, using the previous observation (i.e., test) as the identification key (Fig. 1B). Two different scenarios may arise (see Fig. 1C): (1) functional networks of the same structural network have high similarity between them but a low one compared with the rest, which indicates that the system is *identifiable* (Fig. 1C, first case) or (2) functional networks of the same structural network have the same level of similarity as compared to functional networks from other structural networks, which is the signature of an *unidentifiable* system. Note that in the latter case, the reason can be either all functional networks have a very low similarity between them (Fig. 1C, second case) or, on the contrary, the similarity is very high in all cases, even when comparing two functional networks obtained from different structures (Fig. 1C, third case).

### Experimental setup

**Figure 2:**
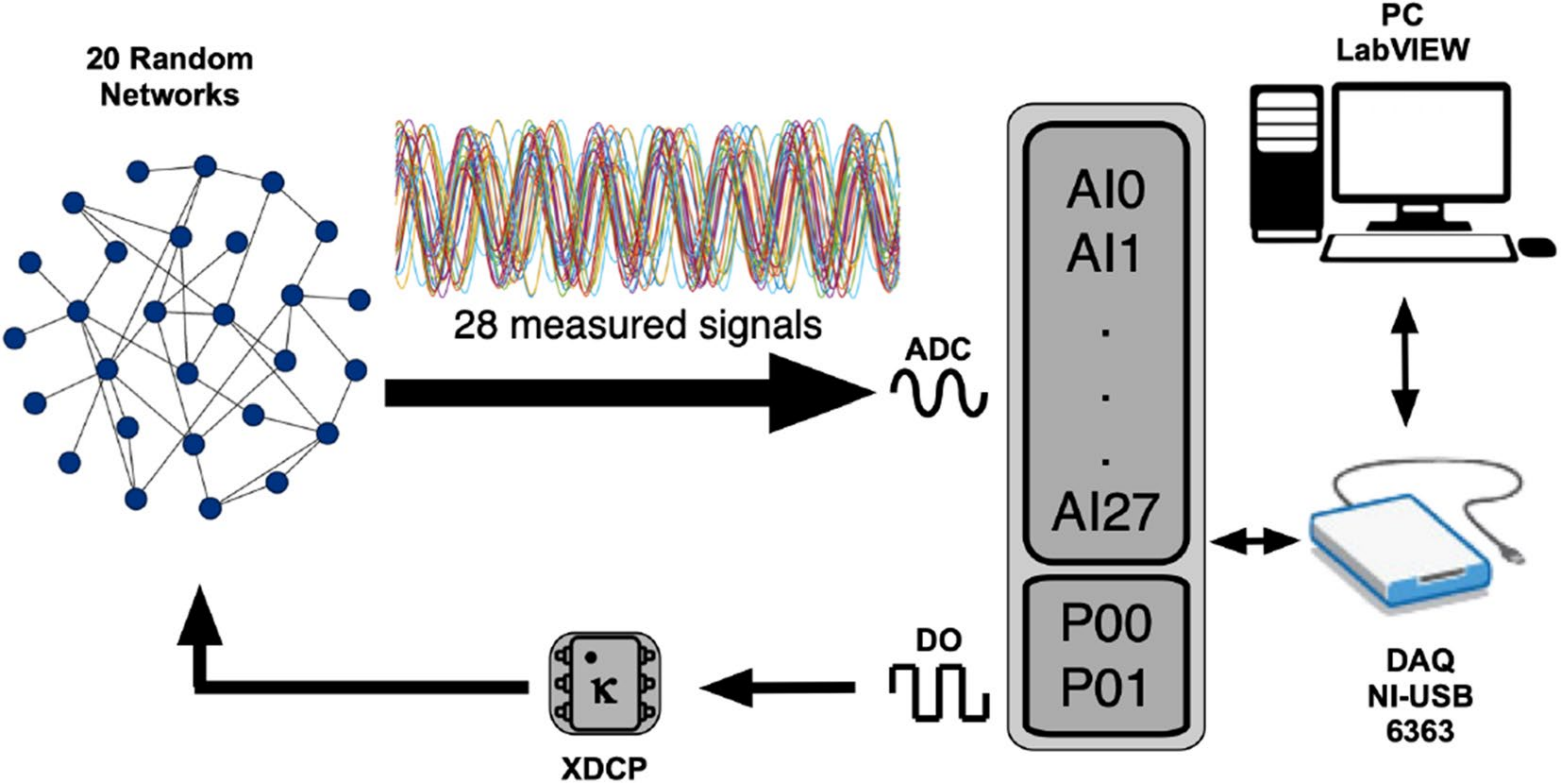
Experimental setup. Schematic representation of the experimental arrangement of a network containing $N=28$ electronic Rössler oscillators. The coupling strength between oscillators ($\kappa$) is adjusted by means of digital potentiometers X9C103 (XDCP), whose resistance is controlled through digital pulses sent by a DAQ (model NI USB 6363, from National Instruments). Port P0.0 is used to increase or decrease the resistance of the digital potentiometer, and port P0.1 sets the initial value (allowing for 100 discretized values of $\kappa$).

We carried out a series of experiments using nonlinear electronic circuits to test how identifiability is related to the level of synchronization of a functional network and its robustness against noise. We constructed $M=20$ structural networks composed of $N=28$ diffusively coupled electronic Rössler oscillators^24^. Equations of the dynamical systems, together with the values of their electronic components (Table 1) and schematic diagrams (Figs. 7, 8) are detailed at “Methods” section. Rössler oscillators were set to have chaotic dynamics in order to better observe the transition from unsynchronized to fully synchronized dynamics. All structural networks had the same number of nodes and degree distributions. The only difference is that we reshuffled the connections between oscillators in order to have the 20 different structures (see “Methods” for details about the structural networks). Figure 2 has a schematic description of the experiment. We used an electronic array (EA), a personal computer (PC), a data acquisition card (DAQ) composed of 28 analog-to-digital converters (ADCs) and 2 digital-to-analog converters (DACs) to record and control the dynamics of the networks. The EA comprised the 28 Rössler electronic oscillators and their corresponding electronic couplers, which sent the dynamics of the oscillators to their outgoing neighbors and, at the same time, collected the inputs from their incoming neighbors. The 28 analog ports (AI0–AI27) acquired the signal of each oscillator. The coupling strength $\kappa$ of the whole network was controlled by two digital potentiometers (XDCP), which were tuned by the signals coming from digital ports P0.0 and P0.1 (DO). During the experiment, we did not add noise to the oscillators, since it will be included directly in the functional networks.

### Estimating differential identifiability

For each of the $M=20$ structural networks, we recorded test and retest dynamics (two acquisitions) of the $N=28$ oscillators for coupling strength configurations ranging from a coupling strength $\kappa =0$ to a value of $\kappa =1$. This guaranteed achieving synchronization of the ensemble for all structural networks. For each value of $\kappa$, we obtained the corresponding two functional networks (test and retest) of a given structural network by computing the phase synchronization between each pair of oscillators. For each node *j*, the instantaneous phase $\phi _j(t)$ was calculated from the Hilbert transform^25,26^ of the time series of its variable $v_2$ (see equations at the “Methods” section for the definition of the Rössler variables). Next, we quantified the phase synchronization between phases $\phi _j(t)$ and $\phi _k(t)$ each pair of oscillators *j* and *k*, obtaining the phase synchronization between the two oscillators as $w_{j,k}=|e^{i[\phi _j(t) -\phi _k(t)]}|$, where $| \cdot |$ indicates temporal averaging. Finally, the average synchronization of the whole network was obtained as $r=\frac{1}{N(N-1)}\sum _{i,j} w_{j,k}$, where $j \ne k$.

For each coupling strength $\kappa$, the values of $w_{j,k}$ were used as the elements of the weighted adjacency matrix $\mathbf{A}(m,\kappa )$ associated with each functional network, with $m=1, 2, \ldots , 20$ being the number of the underlying structural network. We repeated the experiment to have a test and re-test set of functional networks, named $\mathbf{A}(m,\kappa )$ and $\mathbf{A}^*(m,\kappa )$, respectively. On the other hand, we also obtained the adjacency matrices of the underlying structural networks $\mathbf{S}(m)$, which were independent of the coupling parameter $\kappa$.

Once the test and re-test functional networks were obtained, we calculated, for each coupling strength *k*, the Pearson correlation coefficient $p_{ij}$ between all pairs *i* and *j* of the set of *M* functional networks, where *i* belongs to the “test” functional networks and *j* to the “re-test” ones. Finally, we constructed the identifiability matrix ${\mathcal {I}}$, which consists of a $M \times M$ matrix whose elements are directly the values of $p_{ij}$. Note that ${\mathcal {I}}$ is symmetric since $p_{ij}=p_{ji}$. Also note that the *m* element of the matrix diagonal (i.e., $i=j=m$) contains the Pearson correlation coefficient of the structural network *m* when the “test” and “re-test” functional networks are compared, quantifying how similar functional networks are for a given structure.

The identifiability matrix ${\mathcal {I}}$ contains useful information about how reproducible the functional network of a given structure is and, at the same time, how different it is from the functional networks supported by other structures. Therefore, we calculated the self-identifiability $I_{self}$ as the average of the values of diagonal of ${\mathcal {I}}$, which is an indicator of how similar functional networks of a given structure are when they are re-tested. We also obtained $I_{others}$, which is the average of the off-diagonal elements of the identifiability matrix. In this case, $I_{others}$ measures how similar functional networks obtained from two different structures are (in average). Note that the lower $I_{others}$, the more identifiable a structure is within the set of *M* different structures.

Finally, we obtained the *differential identifiability* $I_{diff}$ by comparing how different is $I_{self}$ from $I_{others}$^12^:

1 $$\begin{aligned} I_{diff}= (I_{self}-I_{others}) \times 100 \end{aligned}$$

The differential identifiability $I_{diff}$ indicates to what extent it is possible to distinguish a given network structure from a set of *M* networks just by analyzing the organization of their corresponding functional networks. From now on, the differential identifiability $I_{diff}$ will be our indicator of the systems’ identifiability.

### Functional vs. structural networks in a noisy scenario

**Figure 3:**
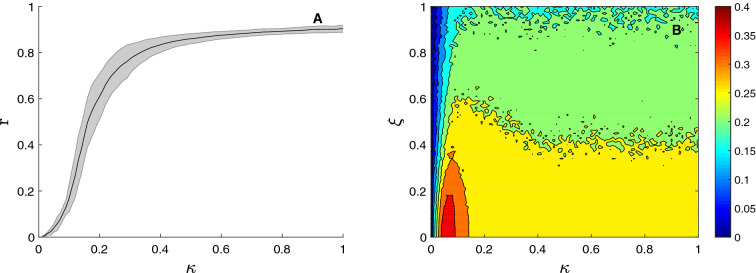
Interplay between functional and structural networks. In (**A**), the average phase synchronization *r* of the whole set of structural networks is plot as a function of the coupling strength $\kappa$. The shadowed region is bounded by the standard deviation of the values of *r*, with the solid line being the average. In (**B**), the average correlation between functional and structural networks $Co(\mathbf{A}^{noise},\mathbf{S})$, also called structural-functional correlation, is plot as a function of the coupling parameter $\kappa$ and the level of noise of the functional networks $\xi$.

Previously to the identifiability analysis, we investigated the interplay between functional networks and their underlying structures. Figure 3A shows the average synchronization parameter *r* of all structural networks as a function of the coupling parameter $\kappa$. We can observe a smooth path to synchronization as $\kappa$ is increased. The shadowed region of Fig. 3 indicates the standard deviation of the values of *r* for all structural networks. As we can see, the largest standard deviation is reported when *r* has a higher increase, i.e., during the transition from the unsynchronized to the synchronized manifold.

Next, we perturbed the elements of all functional networks with a (Gaussian) white noise term of amplitude $\xi$, in order to obtain a set of noisy functional networks $\mathbf{A}^{noise}(m,\kappa ,\xi )$. In such a way, we are accounting for the unavoidable presence of noise that exists in real experiments, which has consequences on the estimation of functional networks, as it is the case, for example, of brain^27^ or molecular^28^ networks. We have two reasons for introducing noise into the elements of the functional network and not directly into the electronic circuits. On the one hand, we did not have access to 28 electronic noise generators (one for each oscillator). On the other hand the consequences of introducing noise directly into the time series are difficult to be interpreted. The reason is that we are concerned about the organization of functional networks and not the dynamics in them. Increasing the noise introduced into the oscillators does not linearly change the structure of the functional networks. In fact, the effects of noise are highly nonlinear and depend on the kind of dynamical system we are implementing and the number of connections of each particular node. For this reason, we preferred to modify directly the elements of the functional matrix. In this way, we assure that the organization of the functional network is changing as the values of the noise amplitude are increased. Furthermore, the effects of noise on functional networks will not depend on the kind of metric (phase synchronization) we used to quantify synchronization. In Fig. 3B, we plot the results of calculating the correlation $Co(\mathbf{A}^{noise},\mathbf{S})$ between the matrices of the noisy functional networks $\mathbf{A}^{noise}(m,\kappa ,\xi )$ and the underlying structural ones $\mathbf{S}(m)$. We can observe how there exists a region of values of $\kappa$ where the correlation between functional and structural matrices is maximized. This is the most convenient scenario to estimate the network structure based on the observation of its dynamics. Note, that this region arises for relatively low values of the coupling strength $\kappa$ (around $\kappa \sim 0.075$). The reason is that high values of $\kappa$ lead to a high synchronization of the majority of oscillators of the network, no matter if they are structurally linked or not, introducing spurious functional links in the estimation. As a consequence, the identification of structural networks based on the observation of node dynamics relies on the existence of a partially incoherent state of the system.

Concerning the effect of the noise amplitude $\xi$, we can observe an impairment of the correlation in all cases. However, it is always around $\kappa \sim 0.075$ where the correlation is higher compared to other coupling strengths.

### Identifiability vs. noise and coupling strength

**Figure 4:**
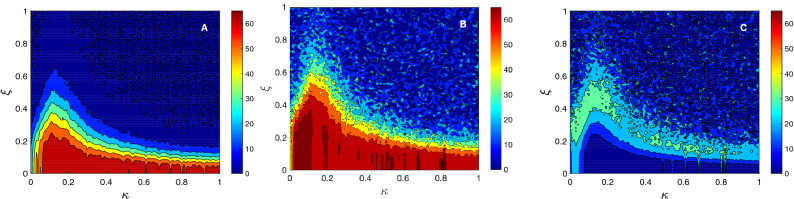
Identifiability as a function of the coupling strength $\kappa$ and the noise amplitude $\xi$. In (**A**), the differential identifiability $I_{diff}$ is plot vs. $\kappa$ and $\xi$. In (**B**), we apply PCA to obtain $I_{diff}^{PCA}$. Finally, in C), we plot the improvement of identifiability when PCA is applied, which is measured as $I_{diff}^{PCA} - I_{diff}$.

But, how does the identifiability of the group of networks depend both on the coupling strength and the level of noise? To answer this question, we show in Fig. 4A the differential identifiability $I_{diff}$ of the *M* networks as a function of the coupling parameter $\kappa$ and the level of noise $\xi$. We can observe that $I_{diff}$ is high for scenarios with low noise amplitudes, and it is completely lost for high noise levels (in this particular case, for $\xi > 0.6$). Concerning the dependence on the coupling parameter, Fig. 4A shows that when $\kappa$ was increased from zero, $I_{diff}$ increased monotonically until reaching a maximum around $\sim 0.15$. If coupling was increased above this value, identifiability decreased again. The reason is that, as we have seen in the previous section, high values of coupling led to a scenario close to the complete synchronization of the functional network, introducing a high amount of spurious functional links, which, in turn, resulted on very homogeneous networks, no matter what the underlying structure is. As a consequence, all functional networks looked similar, and it was difficult to distinguish them.

Going back to $\kappa \sim 0.15$, we can see that, around this value, the network identifiability was especially robust against noise. Only when $\xi > 0.2$, $I_{diff}$ began to decrease, having a reduction around $50\%$ when $\xi \sim 0.4$. If we revisit Fig. 3A, we can observe how this particular value of coupling ($\kappa \sim 0.15$) was close to the region where networks were beginning to synchronize, having low values of the order parameter *r*. Therefore, as it happened in the case of inferring the network structure from the observation of its dynamics, low values of the coupling parameter were the most adequate to promote, in this case, the identifiability of structural networks based on their dynamics.

### Identifiability is increased using the differential identifiability framework

It is worth noting that identifiability strongly depends on the way of quantifying the similarity between functional networks. Regarding to this point, Amico et al.^12^ demonstrated that introducing principal component analysis (PCA) before comparing functional networks reduced the effect of the intrinsic noise of the system, increasing the level of identifiability. Therefore, we followed the same methodology as^12^ and compared each pair of functional networks using only the set of the *l* principal components that maximized the correlation between functional networks. The procedure was as follows: (1) for each coupling strength $\kappa$ assessed, we carried out a group-level PCA decomposition including all test/retest functional networks as obtained from the $M=20$ structural networks (hence the dimensionality of the data and the number of principal components being $2M=40$), (2) we calculated $I_{diff}(l)$ between the matrices containing only the first *l* components (in descending order of explained variance), with $1 \le l \le 2M$, and (3) we identified the number of principal components $l_{max}$ that maximized the value of $I_{diff}(l)$ and hence uncover more functional fingerprints of the circuits. In this way, we obtained the *optimal differential identifiability* as $I_{diff}^{PCA}= max(I_{diff}(l))$, with $l=1,2,\ldots ,2M$ being the number of principal components. Using this methodology, we can asses the levels of differential identifiability achieved as we include more and more components and find the optimal number where $I_{diff}$ is maximum. This raises the following question: are we effectively improving the identifiability of this set of circuit-based structural networks? Results shown in Fig. 4A show $I_{diff}$ values on the original data (i.e. no PCA decomposition/reconstruction applied) for a wide range of coupling strength $\kappa$ and noise amplitude. Figure 4B shows the analogous assessment when reconstructing the functional networks, at each ($\kappa , \xi$) configuration with the number of components that maximized $I_{diff}$ (i.e., $I_{diff}^{PCA}$). It can be observed that the qualitative behavior was similar to the results obtained on the original data. However, both the maximum value $I_{diff}^{PCA}$ and the region where this value was achieved were larger. For couplings close to $\kappa \sim 0.15$, high values of differential identifiability were maintained even for regimes of moderate noise ($\xi \sim 0.4$).

Figure 4C combines both results by calculating the improvement ($I_{diff}^{PCA} - I_{diff}$) achieved when applying PCA. Interestingly, the highest improvement corresponded to regions of moderate noise where, at the same time, the amount of coupling was low.

**Figure 5:**
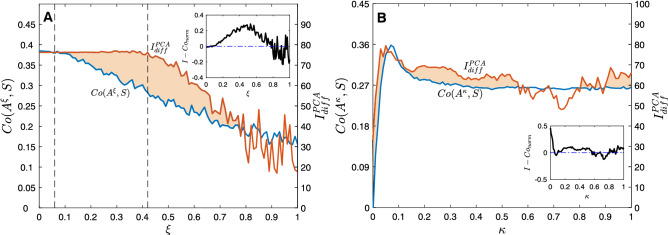
Synchronization vs. identifiability. In (**A**), we show the correlation between the structural and functional networks $Co(\mathbf{A}^{\xi },\mathbf{S})$ (blue line, values on the left vertical axis) and the optimal differential identifiability $I_{diff}^{PCA}$ (orange line, values on the right vertical axis) as a function of the noise amplitude $\xi$ for a fixed coupling parameter $\kappa =0.075$. In the inset (**A**), we plot the differences between the normalized values $I_{norm}-Co_{norm}$. Dashed lines indicate the values of $\xi$ at where $Co(\mathbf{A}^{\xi },\mathbf{S})$ and $I_{diff}^{PCA}$ begin to decrease significantly. In (**B**), we plot the same variables as a function of the coupling strength $\kappa$. In this case, we set the noise amplitude to $\xi =0.18$.

Finally, we investigated the interplay between inferring structural networks from the observation of their dynamics and the differential identifiability. As we have seen in Fig. 3B, the highest correlation between the structural and functional networks $Co(\mathbf{A}^{noise},\mathbf{S})$ was obtained for a coupling parameter $\kappa \sim 0.075$. In Fig. 5A we chose this particular value of $\kappa$ and plot (1) the correlation between the structural–functional networks and (2) the optimal differential identifiability as a function of the noise amplitude $\xi$, respectively $Co(\mathbf{A}^{\xi },\mathbf{S})$ and $I_{diff}^{PCA}(\xi )$. Vertical dashed lines indicate the value of noise amplitude at which both the structural–functional correlation and the optimal identifiability began to decrease. Note how the structural–functional correlation is more affected by low-to-moderate noise amplitudes, beginning its declining much faster than identifiability, which held its initial value up to $\xi \sim 0.4$. We normalized both parameters dividing their values by their means, obtaining the normalized identifiability $I_{norm}=\frac{I_{diff}^{PCA}(\xi )}{ \langle I_{diff}^{PCA} \rangle }$ and the normalized structural–functional correlation ($Co_{norm}=\frac{Co(\xi )}{ \langle Co(\xi ) \rangle }$). Next, we calculated $I_{norm}-Co_{norm}$. The inset of Fig. 5A shows how the maximum difference between both normalized parameters corresponded to intermediate noise amplitudes ($\xi \sim 0.5$). In other words, for situations with intermediate values of noise, the correlation between structural and dynamical networks decreases, however, the identifiability parameter better maintains the values reported without noise sources. Figure 5B shows the behaviour of the same variables as a function of the coupling strength $\kappa$. In this case, we set the noise amplitude to $\xi =0.18$ and analyze how the correlation $Co(\mathbf{A}^{\kappa },\mathbf{S})$ between the structural and functional networks changes $Co(\mathbf{A}^{\kappa },\mathbf{S})$, paying attention at the same time to the value of $I_{diff}^{PCA}(\kappa )$. We observe how $I_{diff}^{PCA}(\kappa )$ overcomes $Co(\mathbf{A}^{\kappa },\mathbf{S})$ for a wide range of coupling strengths. Also note that very low and very high values of $\kappa$ have negative consequences on (1) the correlation between the structural and functional networks and (2) identifiability. In this way, a maximum appears around $\kappa =0.05$ for both variables. As we can observe in Fig. 5B, both peaks are very close to each other. However, the peak of the inedibility is reached first, i.e., lower coupling strengths are needed to achieve the highest identifiability. Furthermore, identifiability better resists the inconveniences of increasing the coupling strength after the peak. This fact can be better observed at the normalized values $Co_{norm}=\frac{Co(\kappa )}{ \langle Co(\kappa ) \rangle }$ and $I_{norm}=\frac{I_{diff}^{PCA}(\kappa )}{ \langle I_{diff}^{PCA} \rangle }$, whose difference is plot in the inset of Fig. 5B. $I_{norm}-Co_{norm}$ reveals that the normalized identifiability is higher than the structural–functional correlation for moderate values of the coupling strengths ($0.1< \kappa < 0.56$).

## Discussion

We quantified the identifiability of a set of structural networks based on the observation of the dynamics of their nodes. First, we have seen how different network structures led to a different set of functional networks when the coupling parameter between the nodes of the network was increased. Importantly, when networks were completely unsynchronized, the resulting functional networks had a low number of links, whose weights were, in turn, low. As a consequence, the correlation with the underlying structural network was low. On the other hand, when the coupling parameter was high, it was possible to reach the synchronized state, leading to fully connected functional networks and, as a consequence, to a low correlation between structural and functional networks. We have seen that it was for low coupling strengths when the highest correlation between a network structure and its corresponding functional network was the highest. Next, we studied how the presence of noise in the construction of functional networks deteriorates the correlation between structure and function.

Fortunately, it is in scenarios with intermediate values of noise where we have seen that the identifiability parameter is more robust. This parameter indicates if it is possible to distinguish between different network structures just by looking at their dynamics. Suppose we had a given set of network structures and a sample of their corresponding functional networks. Now, someone provided us with a new mysterious functional network without indicating what its underlying structure was. If a system were identifiable, we would be able to infer what network structure corresponded to the new functional network just by comparing it to the complete set of the previous functional networks. However, as in the case of inferring structural links from node dynamics, we observed that identifiability is highly dependent on the coupling parameter. Very high coupling strengths led to fully connected functional networks hindering to find differences between functional networks obtained with different structures. It is at low coupling parameters (i.e., for regions of low synchronization) where identifiability is the highest since functional networks are more heterogeneous and, at the same time, more correlated with the underlying structures. In the absence of noise, identifiability was maintained for a wide range of couplings. Interestingly, we observed how, in the presence of noise, identifiability was quite robust, especially when PCA was introduced to quantify the similarity between functional networks.

It is worth mentioning the limitations of the identifiability parameter. First, we need to have previous knowledge of how many different systems (i.e., structural networks) we are dealing with. Second, we need previous recordings of the dynamics of each structural network to quantify the matching between each structural and functional network. These two requirements seem to be too strong in certain real systems, but not all of them. For example, the identifiability parameter is being used to analyze brain imaging datasets. In this application, the brain activity of a group of individuals is recorded using a different brain imaging technique, such as fMRI, EEG, or MEG. Next, functional networks are constructed from the activity of each brain region. Note that, in this case, the underlying structure of the brain networks is not known, since it is not possible to have a complete reconstruction of the anatomical network of each individual. However, it has been demonstrated that individuals can still be identified thanks to the methodology we have followed in the current work, which opens the door to a diversity of future applications.

Finally, two key points must be stressed concerning the applicability of the identifiability parameter. First, identifiability depends on the specific set of structural networks that we want to distinguish. Therefore, a given structure can be identifiable when compared within a group of networks with entirely different structures, but the same structure could be unidentifiable when the rest of the members of the group are similar. Further studies should clarify the minimal structural differences between networks to make them identifiable. Second, our results are highly dependent on the dynamical system placed on the nodes of the networks, and it should be investigated whether the results obtained here can be extended to other kinds of dynamics systems.

Given all, we believe that identifiability can be a useful indicator to distinguish between systems whose dynamics are strongly dependent on its structure and those that are not. The dynamics of the former would be adequate candidates to infer the underlying structural properties of the system, in case we do not have access to its wiring connections. On the contrary, inferring the structural networks of systems with low identifiability could be a much harder task.

## Methods

### Network dynamics: the Rössler oscillator

Equations describing the dynamics of the electronic Rössler oscillators are^24,29,30^:

2 $$\begin{aligned}&{\dot{v}}_{1i}=-\frac{1}{R_{1}C_{1}}\left( v_{1i}+ \frac{R_{1}}{R_{2}}v_{2i}+\frac{R_{1}}{R_{4}}v_{3i}\right) \end{aligned}$$

3 $$\begin{aligned}&{\dot{v}}_{2i}=-\frac{1}{R_{6}C_{2}}\left( -\frac{R_{6}R_{8}}{R_{9}R_{7}}v_{1i}+ \left[ 1- \frac{R_{6}R_{8}}{R_{c}R_{7}}\right] v_{2i}-\kappa \frac{R_{6}}{R_{15}}\sum _{j=1}^{N}{a_{ij}(v_{2j}-v_{2i})}\right) \end{aligned}$$

4 $$\begin{aligned}&{\dot{v}}_{3i}=-\frac{1}{R_{10}C_{3}}\left( -\frac{R_{10}}{R_{11}}G\left( v_{1i}\right) +v_{3i}\right) \end{aligned}$$

where ${v}_{1i}$ , ${v}_{2i}$ and ${v}_{3i}$ are the three voltages that describe the dynamical state of oscillator *i*, *R* and *C* are resistances and capacitors, $\kappa$ is the coupling strength (controlled by a digital potentiometer) and $a_{i,j}$ are the elements of the adjacency matrix **A**, with $a_{ij}=1$ if the output of oscillator *i* is used as the input of oscillator *j* and zero if both circuits are not connected in the direction $i \rightarrow j$. Finally, $G(v_{1i})$ is a piecewise nonlinear function given by:

5 $$\begin{aligned} G(v_{1i})= \left\{ \begin{array}{lcc} 0 &{} if &{} v_{1i} \le V_d+V_d \frac{R_{14}}{R_{13}}+V_{ee}\frac{R_{14}}{R_{13}} \\ \\ \frac{R_{12}}{R_{14}}v_{1i}-V_{ee}\frac{R_{12}}{R_{13}} -V_d\left( \frac{R_{12}}{R_{13}}+\frac{R_{12}}{R_{14}} \right) &{} if &{} v_{1i}> V_d+V_d \frac{R_{14}}{R_{13}}+V_{ee}\frac{R_{14}}{R_{13}} \\ \end{array}\right. \end{aligned}$$

### Structural networks

**Figure 6:**
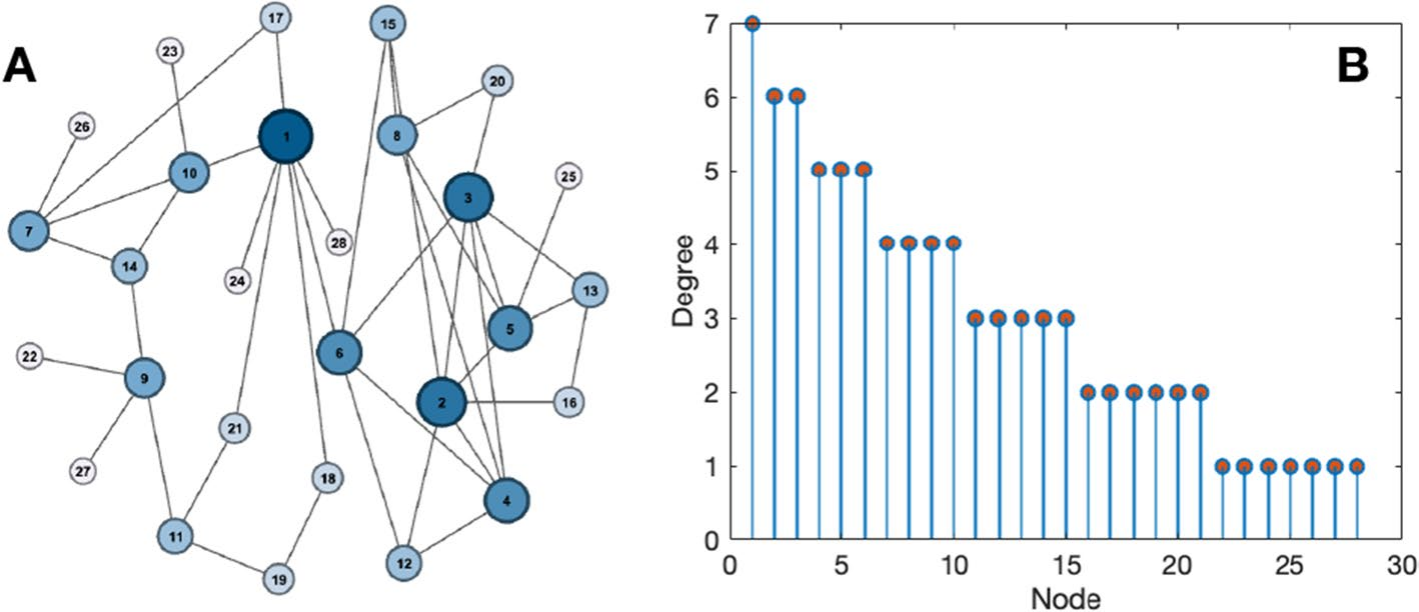
Structural networks. (**A**) Example of one network structure. (**B**) Node number vs. its degree of all structural networks.

We constructed $M=20$ different structural networks, all of them composed of $N=28$ nodes (see Fig. 6A for an illustrative example). We used the same degree distribution for all networks, trying to have a certain amount of heterogeneity in the nodes’ degree (i.e., number of neighbors). Figure 6B shows the precise number of neighbors each node has, with a hub (node $\#$1) having 7 neighbors and, on the opposite side of the distribution, 7 nodes with degree 1. To obtain the set of *M* different networks, we randomly reshuffled the links of each node, with the only conditions of maintaining the total number of neighbors and avoiding self-loops. Furthermore, we kept the node number along with the structural networks and, therefore, node *i* (with $i=1,2,\ldots ,N$) always had the same number of links in all the different structures. We calculated the shortest path length *d* and the clustering coefficient *C* of the structural networks, obtaining an average of $\langle d \rangle = 2.92$ and $\langle C \rangle = 0.08$ and a standard deviation within the group of $d_{std}=0.10$ and $C_{std}=0.04$, respectively.

### Parameters of the electronic components

We set the parameters of Eqs. (2)–(4) in order to have chaotic dynamics at the Rössler oscillators. The main advantage of being in such a regime is that the order parameter *r* is a good indicator of the global synchronization of the network, which allows relating the reported values of the identifiability parameter with the level of synchronization of the whole network. The specific values of the electronic components are summarized in Table 1.

**Table 1:** Parameters of the Rössler electronic oscillator in the chaotic regime.

C1 = 1nF	C2 = 1nF	C3 = 1nF	$\kappa = [0,1]$
$R_{1}=2\,M\Omega$	$R_{2}=200\,k\Omega$	$R_{3}=10\,k\Omega$	$R_{4}=100\,k\Omega$
$R_{5}=50\,k\Omega$	$R_{6}=\,5M\Omega$	$R_{7}=100\,k\Omega$	$R_{8}=10\,k\Omega$
$R_{9}=10\,k\Omega$	$R_{10}=100\,k\Omega$	$R_{11}=100\,k\Omega$	$R_{12}=150\,k\Omega$
$R_{13}=68\,k\Omega$	$R_{14}=10\,k\Omega$	$R_{15}=500\,k\Omega$	$Rc= 58\,k\Omega$
$V_{d}=0.7$	$V_{ee}=9$		

### Circuit diagrams

#### The Rössler oscilator

**Figure 7:**
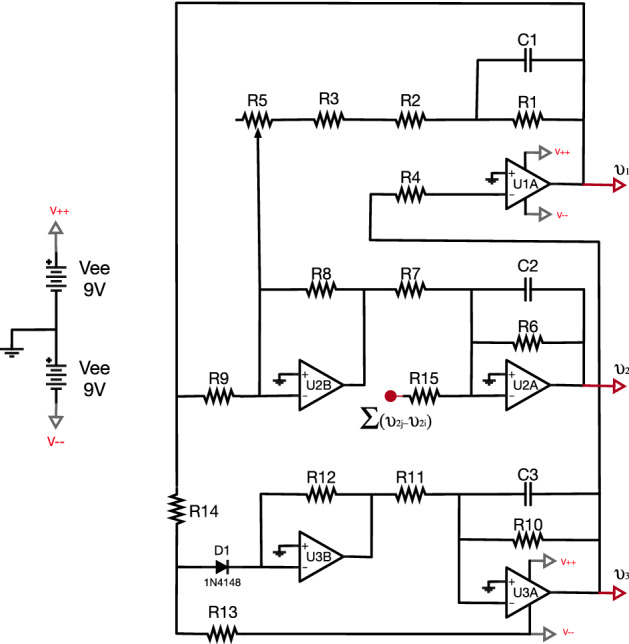
Electronic representation of the Rössler oscillator.

We use the Rössler system described in Ref.^30^, which is composed of a combination of resistances, capacitors, diodes and operational amplifiers (Op-Amp). All parameters are fixed and equal to all oscillators. Figure 7 contains the circuit diagram.

#### The coupler circuit

**Figure 8:**
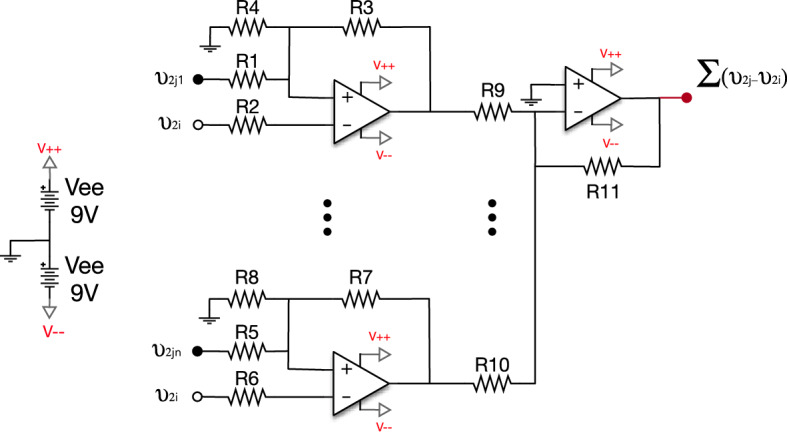
Electronic representation of coupler circuits. This circuit works as a diffusive coupling between an electronic oscillator and its neighbors. Each differential Op-Amp receives the output of oscillator *j*, which is used as the input of oscillator *i*. Another Op-Amp (the one on the right) adds the inputs of all the incoming signals of oscillator *i* that are received from its *j* neighbours. All resistances of the coupler circuit have a value of $R_i=1\,k\Omega$.

Each node of the network (i.e., Rössler oscillator) is connected to its neighbours according to the adjacency matrix $\mathbf{A}$. As shown in Eq. (3), the coupling between a node *i* and its neighbor *j* is diffusive ($v_{2j}-v_{2i}$). Next, all inputs of node *i* are added using an Op-Amp in the voltage adder configuration. See Fig. 8 for details about the circuit diagram.
